# Case report of an unexpected inflammatory myofibroblastic tumor

**DOI:** 10.1016/j.amsu.2021.102316

**Published:** 2021-04-22

**Authors:** Vishal Farid Raza, Dawood Arshad, Sajeel Ahmad, Khalid Javeed Khan

**Affiliations:** Department of General Surgery, Sir Ganga Ram Hospital, Lahore, Fatima Jinnah Medical University, Queen's Road, Lahore, 54000, Pakistan

**Keywords:** Case report, Spleen, Inflammatory myofibroblastic tumor

## Abstract

**Introduction:**

Inflammatory myofibroblastic tumor is a benign entity that may present as a locally aggressive malignancy, predominantly in the lung. 500 cases have been reported in the literature, and an estimated prevalence ranges from 0.04% to 0.7%.

**Case presentation:**

An eighteen-year old male presented to the surgical clinic with abdominal pain. The pain was recurring despite trials of analgesics and remained undiagnosed. Radiological imaging demonstrated a lesion in the spleen. An infectious cause was presumed due to their endemicity in South Asia, however pathology showed an inflammatory myofibroblastic tumor.

**Discussion:**

Pre-operative imaging is yet to develop a set criterion that may identify this lesion, though clinicians may be clued in by the benign appearance despite the large size. Usual diagnosis is on pathology after complete surgical excision, which is the mainstay treatment advocated.

**Conclusion:**

Reporting of the tumor in unusual sites such as the spleen is scarce, increase of which may help establish guidelines, understand tumor behavior and guide clinicians that may encounter it in surgical practice.

## Highlights

• IMTs may present in unusual sites such as the spleen and be seemingly aggressive for a benign tumor.
• Splenic masses or masses anywhere that have an hypodense pattern with late contrast enhancement should be suspected for inflammatory myofibrolastic tumors.
• In developing countries with endemic infectious diseases IMTs may resemble tuberculosis or hydatid cyst disease.
• EBV and anaplastic lymphoma kinase gene testing should be undertaken for these tumors.

## 1 Introduction

**Fig. 1:**
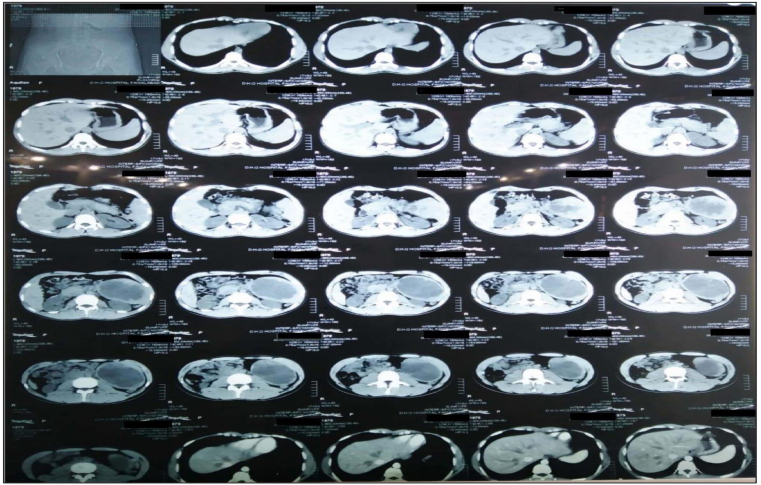
Computed Tomography scan of the abdomen with intravenous contrast enhancement showing the splenic patholog*y*.

**Fig. 2:**
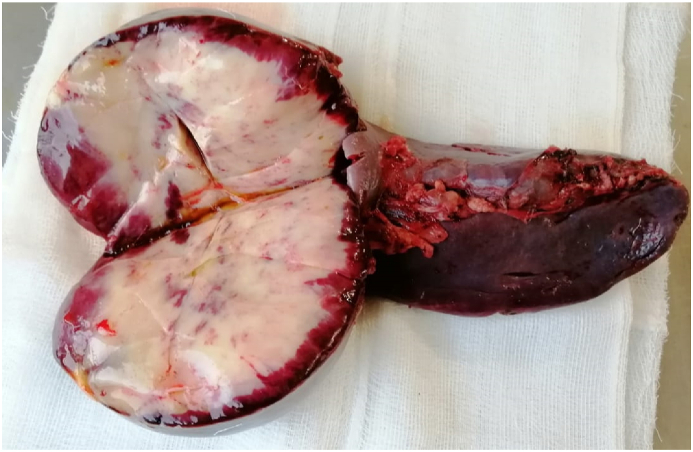
Image of resected spleen incised at the upper pole to examine gross pathology post-operatively.

Inflammatory myofibroblastic tumor has been known by many names, including pseudosarcoma and inflammatory psuedotumor. It is characterised by spindle shaped cells with inflammatory cells in the connective tissue. Its biological behavior is largely undefined and considered benign, however recurrences and metastasis have been cited in the literature [1]. When malignant behavior is present less than 5% cases having metastasis and of all the cases 8–18% may have malignant transformation. Myofibroblastic tumors seem to predominantly affect the lung however they may occur in any organ system, with a low prevalence ranging from 0.04% to 0.7%, affecting mainly younger individuals [2,3]. 500 cases have been reported in the literature, and from these the role of Anaplastic Lymphoma in cytogenetic analysis has been described, which has fuelled the current debate of whether it is truly a pseudotumor or a low grade true tumor [4]. In our setting the Case report was interesting due to the prevalence of infectious diseases in Pakistan, a splenic mass was more likely to be due to tuberculosis or hydatid cyst disease. Until pathological assessment neither surgical nor radiological differentials included the possibility of an inflammatory myofibroblastic tumor. We report this case in hopes it may aid clinicians from developing countries to broaden their pre-operative differential diagnosis. (see Fig. 1, Fig. 2)

## 2 Case presentation

This Case report was reported in line with the SCARE 2020 criteria [5]. An 18 year old South Asian male presented to the surgical clinic, with complaints of generalised abdominal pain for 3 months. The pain was dull, occurring at any time of the day with no definite pattern. Over the counter analgesics alleviated his pain. Exacerbating factors were not identified His pain did not hinder mobility of daily functioning.

On a review of systems he had intermittent diarrhea responsive to symptomatic treatment. He exprienced no weight loss. He had no history of past medical illneses or surgery. His family history was unremarkable, he was a student and belonged to a low-middle income household. He was a non smoker, with no substance use history, and had no congenital or inhertiable diseases present in the family.

On examination, his abdomen was flat, with slight protuberance on the left hemiabdomen. No cutaneous manifestations were visible. On palpation, there was a firm mass palpable from the umbilicus to the hypogastrium and extending laterally till the left lumbar region.The mass was mildly tender on palpation. The mass did not move with respiration and there was no bruit on auscultation. Bowel sounds were audible and normal. General physical examination and systemic examination besides abdominal examination were unremarkable.

His visits to general physicians for pain spanned over a period of 8 months, and resulted merely in symptomatic treatment. Due to delay in investigation he presented late for definitive care.

A Computed Tomography scan of the abdomen was done on his own initiative after non resolution of pain.

The secondary care hospital in his locality referred him to a tertiary care center, following which he presented to us.

We began work up suspecting hydatid cyst disease based on his CT scan findings.

An ultrasound was done at our center to corroborate the CT scan findings.

Vaccination for pneumoccocus, meningococcus and haemophilus influenzae were done and he was planned for an elective interval splenectomy.

### 2.1 Investigations

Baseline investigations showed a CBC within normal limits with no eosinophilia, and an ESR of 30.

Liver functions tests, renal function tests and electrolytes were within normal limits.

Echinococcal indirect haemagluttination test was negative.

Ct scan reported*: “*a moderately enlarged spleen with cystic lesion of 11 × 10cm at its lower pole, with enhancing walls. Left kidney is slightly inferiorly displaced. Lymph nodes noted in mesentery, largest measuring 10mm. No focal lesions elsewhere. Features suggestive of splenic abscess or hydatid cystic disease.”

Ultrasound reported: “an echogenic mass of 8.8x9.3 × 6.3cm size at lower pole of spleen having a volume of 269ml with well circumscribed walls. The lesions is more dense centrally with few echogenic septa visible. No definite scolices or fluid seen. Features consistent with thick debrinous calcified hydatid cyst in the spleen.”

A chest X-ray was done which was unremarkable.

### 2.2 Outcome

He underwent a laparoscopic assisted splenectomy in January 2020 done by the Professor of Surgery of the department. The abdomen was approached laparoscopically and the spleen was mobilised and the pedicle ligated. After splenectomy, due to the size of the spleen and the need for pathological assessment, a 5cm upper midline incision was given to deliver the spleen. A 6 × 7cm solid mass was identified in the upper pole of the spleen confined to the spleen, not breaching the capsule grossly. It was a hard mass and there was no hydatid cyst disease visible to the naked eye.

Pathology reported an irregular grey-white firm area measuring 9x6x3cm with necrotic areas and congestion, capsule is intact. Histological examination revealed spleen showing massive congestion and hemosiderin laden macrophages. In addition a few areas of coagulative necrosis, and a cellular neoplasm composed of spindle to polygonal cells admixed with edema, lymphocytic and plasma cell infiltrate and extravasated RBCs. Subcapsuar dilated and congested vascular channels. Impression: Inflammatory myofibroblastic tumor with areas of infarction.

An oncologist was consulted who opined that there is no need for any oncological intervention. The patient was followed after discharge a week later, when sutures were removed and wound was healthy. He was then followed monthly for 6 months, and was found to have no issues and was doing well.

## 3 Discussion

Hydatid cyst disease was a possible diagnosis during the patient's work up. It is found where dogs and cattle are. The disease in the liver, represents 65% of cases, pulmonary manifestation may be up to 25%. Symptoms develop as the cyst grows and pressure effects cause pain or discomfort [6]. Pakistan has a prevalence of the diease up to 46%, with 7% of stray dogs carrying echinoccous [7]. A study in Pakistan showed that splenic cases were third most common at 3.5% [8]. On imaging cysts may show calcification in up to 30% of cases, and on unenhanced CT scans high attenuation of cyst wall is common [9] This differential was kept keeping our local endemicity and the radiological findings in sight.

Splenic abscesses are relatively uncommon diagnoses. They may be complications of infective endocarditis in immunocompromised patients. They may represent a contiguous spread from pancreatic abscesses or diverticular disease. The organisms involved are generally polymicrobial. Mortality may reach 70% without drainage and gold standard is splenectomy [10]. Given the high incidence of tuberculosis in Pakistan, a tuberculoid splenic abscess was considered. Though splenic tubercuosis has been reported, abscess formation is rare [11]. Given our local endemicity of tuberculosis a cold abscess presenting as an enlarging mass with pain or discomfort was kept as a differential diagnosis, supported by the radiologist's opinion of a possibility of splenic abscess.

There are only a handful reported cases of splenic IMTs and an interesting series was reported where the authors tested twelve such cases for an association with Epstein Bar Virus, and found that 2 cases had EBV membrane protein identifiable and 10 had latent RNA found in it. An association with EBV, that is a known oncovirus, could explain in part the cytogenetic changes that are being discovered in association with IMTs, however our patient did not have a history of infectious mononucleosis or any other EBV caused diseases, however this does not preclude the possibility that he was asymptomatically infected by the virus [12].

The tumor was first described in 1939, and in the 1980s two cases were first described in the spleen. These two cases were largely incidental findings, one with a mass lesion on CT and one discovered on pathology, much like the usual presentation of IMT, where a lesion requiring surgical excision leads to the discovery of an IMT on pathology. This is important for when a mass lesion is not befitting any other diagnosis, and is presenting in a largely benign manner for a lesion of its size, one should be alert to the possibility of an IMT [13,14].

In our patient, radiologically it seemed to be a calcified hydatid cyst, with septa and central dense area, and cystic areas identified on ultrasound. The literature seems to show that radiologically the most common differentials were either splenic malignancy, or lymphoma. Ultrasound examination usually showed areas of calcification, echogenicity within the spleen which was usually a hyperechoic mass, however this was not uniformly homogenous or heterogenous in each Case. The echogenicity patterns were at most, variable for each mass and non-specific. CT scanning mostly identified low density masses that were either iso or hypodense to the spleen in non contrast enhanced images but showed contrast on enhancement in the delayed phase. Some cases showed calcifications. The CT did usually show a well defined, circumscribed wall or rim around the lesion. However, of note, there are no pre-operative radiological imaging signs that could definitely state that the mass is an IMT, and a presentation on CT scan as that of a calcified hydatid cyst has not been reported in earlier series to our best knowledge [[15], [16], [17], [18], [19], [20], [21]].

Most patients presented with non specific abdominal pain, or fullness in the left hypochondrium. There was one patient who had a mass growing for 9 years, and was compared to a previous CT done 9 years earlier and found to have enlarged [15], similarly ultrasound series have shown the growing size of the mass in patients [16].

There are currently no guidelines as such for the treatment off IMTs however chemotherapy has generally not been advocated, and there are no guidelines for organ specific IMTs. Surgical resection is the preferred mainstay of treatment [16]. However, there are cases of successful treatment with chemotherapy, NSAIDs, steroids, radiation and targeted therapy in different combinations and as individual therapy for unresectable or recurring disease as well in the literature [[22], [23], [24], [25]].

## 4 Conclusion

In conclusion we found that the benign biological behavior is the norm for such tumors, despite their rapid growth rate and discomfort or pain that they might cause. They may be found anywhere and more research is due for IMTs as a clinical entity overall to establish guidelines, and further research is needed using cases reported for site specific behavior so that guidelines may be established for each organ site. Further it is important to remember that when this rare tumor is encountered, EBV testing and anaplastic lymphoma kinase gene testing should be undertaken.

## Provenance and peer review

Not commissioned, externally peer-reviewed.

## Annals of medicine and surgery

The following information is required for submission. Please note that failure to respond to these questions/statements will mean your submission will be returned. If you have nothing to declare in any of these categories then this should be stated.

## Funding

There are no sponsors involved in funding of the research, which is undertaken by the authors themselves.

## Ethical approval

As patient data has been kept anonymous and it is a Case report, departmental approval was sought.

## Consent

Written informed consent was obtained from the patient for publication of this Case report and accompanying images. A copy of the written consent is available for review by the Editor-in-Chief of this journal on request.

## Credit author contribution

Dawood Arshad and Sajeel Ahmad were involved in data collection and interpretation and outlining of the article.

Vishal Farid Raza and Khalid Javeed Khan were involved in writing the paper, study concept designing, editing and proof reading and interpretation of the data.

## Registration of research studies

1. Name of the registry:
2. Unique Identifying number or registration ID:
3. Hyperlink to your specific registration (must be publicly accessible and will be checked):

## Guarantor

Dr. Vishal Farid Raza

vishalraza@hotmail.com.

## Declaration of competing interest

No conflicts of interest present.
